# Efficacy of Osteoporosis Medications for Patients With Chronic Kidney Disease: An Updated Systematic Review and Network Meta-Analysis

**DOI:** 10.3389/fphar.2022.822178

**Published:** 2022-02-11

**Authors:** Chia-Hsien Chen, Wei-Cheng Lo, Ping‐Jen Hu, Hsiu-Chen Chan, Wan-Chen Shen, Mai-Szu Wu, Mei-Yi Wu

**Affiliations:** ^1^ Department of Orthopedics, Shuang Ho Hospital, Taipei Medical University, New Taipei City, Taiwan; ^2^ Department of Orthopedic Surgery, School of Medicine, College of Medicine, Taipei Medical University, Taipei, Taiwan; ^3^ School of Biomedical Engineering, College of Biomedical Engineering, Taipei Medical University, Taipei, Taiwan; ^4^ Master Program in Applied Epidemiology, College of Public Health, Taipei Medical University, Taipei, Taiwan; ^5^ Division of Gastroenterology, Department of Internal Medicine, Shuang Ho Hospital, Taipei Medical University, New Taipei City, Taiwan; ^6^ Master’s Program in Biomedicine, College of Science and Engineering, National Taitung University, Taitung, Taiwan; ^7^ Department of Pharmacy, Shuang Ho Hospital, Taipei Medical University, New Taipei City, Taiwan; ^8^ Division of Nephrology, Department of Internal Medicine, Shuang Ho Hospital, Taipei Medical University, New Taipei City, Taiwan; ^9^ Division of Nephrology, Department of Internal Medicine, School of Medicine, College of Medicine, Taipei Medical University, Taipei, Taiwan; ^10^ Taipei Medical University Research Center of Urology and Kidney, Taipei Medical University, Taipei, Taiwan; ^11^ College of Public Health, Institute of Epidemiology and Preventive Medicine, National Taiwan University, Taipei, Taiwan

**Keywords:** osteoporosis, chronic kidney disease, fracture, bone mineral density, network meta-analysis

## Abstract

**Background:** Chronic kidney disease (CKD) is associated with bone and mineral metabolism. In this study we evaluated the comparative efficacies and safety of osteoporosis medications in patients with CKD or a history of kidney transplantation, and make recommendations for the best choice of osteoporosis treatment among patients with CKD or a history of kidney transplantation.

**Methods:** We systemically searched for randomized controlled trials published in PubMed, Embase, and Cochrane databases up to June 2020. Network-meta analysis was used to compare the relative effectiveness of different treatments. A random-effects model was used when heterogeneity was expected. The safety of different treatments was also evaluated in terms of reported major adverse events.

**Results:** A total of 17 studies with data from 10,214 patients who had stage 2–5 CKD, were receiving dialysis, or had a history of kidney transplantation were included in the network meta-analysis. Treatment with teriparatide, denosumab, alendronate, and raloxifene were all associated with a significantly reduced risk of fractures compared to treatment with placebos [teriparatide: odds ratio (OR) = 0.19, 95% confidence interval (CI): 0.10–0.35; denosumab: OR = 0.40, 95% CI: 0.27–0.58; alendronate: OR = 0.61, 95% CI: 0.40–0.92; raloxifene: OR = 0.52, 95% CI: 0.41–0.67]. The rank probability and the surface under the cumulative ranking (SUCRA) values suggested that teriparatide ranked the highest for improvement in vertebral bone mineral density (BMD) (SUCRA = 97.8%), whereas denosumab ranked the highest for improvement in femoral neck BMD (SUCRA = 88.3%).

**Conclusion:** Teriparatide and denosumab seem to be the most effective treatments for preventing bone loss and reducing the risk of fracture in our network comparison. However, because of the limitations and potential biases in the reviewed studies, there is still some uncertainty about the best treatment options for osteoporosis in patients with CKD or a history of kidney transplantation.

**Systematic Review Registration**: [PROSPERO], identifier [CRD42020209830].

## Introduction

Chronic kidney disease (CKD) is a major global public health issue, affecting an estimated 700 million people worldwide (Bikbov et al., 2020). According to the United States Renal Data System (USRDS) 2020 Annual Data Report, 14.9% of American adults surveyed between 2015 and 2018 had CKD (System URD, 2013). Globally, the prevalence of CKD has almost doubled over the last 2 decades, driven by population growth, aging, and an increased number of people with hypertension and diabetes (Bikbov et al., 2020). The growing number of CKD cases and kidney transplantation may lead to a potential increase in the burden of bone and mineral metabolism disorders. Studies of patients with CKD or a history of kidney transplantation have shown that there is a higher incidence of hip fracture among patients with progressive CKD compared to patients without CKD (System URD, 2013). Furthermore, according to the results of the Dialysis Outcomes and Practice Patterns Study, patients undergoing hemodialysis have higher rates of fracture, death, and hospitalization than the general population (Tentori et al., 2014). Osteoporosis also causes an economic burden, with the total cost per year exceeding that of brain disorders (Pisani et al., 2016).

Currently, there are no standardized treatment recommendations for how to treat osteoporosis among patients with CKD or a history of kidney transplantation. The Kidney Disease Improving Global Outcomes (KDIGO) guidelines were designed to facilitate decision-making in the treatment of patients with CKD or a history of kidney transplantation, however these guidelines do not yet give definitive recommendations for how to best treat mineral and bone disorders (Ketteler et al., 2017). One systematic review compared the safety and efficacy of several different osteoporosis medications for treatment of patients with stage 3–5 CKD or a history of kidney transplantation (Wilson et al., 2017). However, the authors of that review were not able to definitively determine the best osteoporosis medication for patients with CKD due to limited evidence. A network meta-analysis can overcome problems with limited evidence by allowing researchers to compare a network of direct and indirect results from multiple studies. We performed a network meta-analysis to compare various osteoporosis medications and summarized the evidence to develop improved recommendations for the best pharmaceutical treatment options for osteoporosis among patients with CKD or patients with a history of kidney transplantation.

## Materials and Methods

This meta-analysis was performed in accordance with a registered protocol (CRD42020209830). The method of analysis used in this study was consistent with that used in previous published studies (Group KDIGOC-MW, 2009; Wilson et al., 2017).

### Search Strategy

We searched the MEDLINE, Embase, and the Cochrane Central Register of Controlled Trials databases for keywords related to randomized controlled trials comparing the effects of several osteoporosis drugs among patients with severe kidney problems. Severe kidney problems were defined as CKD, a history of receiving dialysis, or a history of kidney transplantation. The drugs we included in our network metanalysis were bisphosphonates, teriparatide, denosumab and raloxifene. We excluded studies that did not compare the osteoporosis drug treatment to a placebo, usual care, or an active control. Studies that compared two of the included drugs to each other without comparing to a placebo, usual care, or an active control were not included. To be consistent with previous studies, the search was limited to English-language human studies conducted between December 2016 and June 2020. We also reviewed the studies included in previous publications (Group KDIGOC-MW, 2009; Wilson et al., 2017). A complete description of our search strategy is detailed in the supplementary materials. A manual search of the references of several relevant studies was also performed to avoid missing any articles.

### Selection Criteria

After exclusion of duplicate studies, two investigators (PJH and HCC) independently screened the titles and the abstracts of studies and evaluated the full texts to determine their eligibility. The investigators resolved disagreements by discussing with a third author (WCL). Studies were required to meet the following criteria for inclusion in our research: 1) the study must be a randomized controlled trial of patients diagnosed as having CKD (stage 3–5), receiving dialysis, or having undergone kidney transplantation; 2) the study must include at least 25 patients and must track patients for at least 6 months after treatment; 3) the study must include at least one pairwise comparison of the interventions listed in the search strategy section; and 4) the study must evaluate bone mineral density (BMD), incident fractures, or adverse events. We excluded animal, *ex vivo*, and toxicological studies as well as duplicates, summaries, commentaries, editorials, case reports, case series, and conference abstracts.

### Data Extraction and Quality Assessment

The following information was extracted from each study: first author, publication year, study characteristics (e.g., location, sample size, funding source), osteoporosis medications (e.g., type of medicine, dosage regimen), patient characteristics (e.g., number of patients, age, sex, and ethnicity), definition of BMD and fracture (e.g., methods of measurement), change in BMD and risk of adverse fractures [e.g., effect size and 95% confidence intervals (CIs)], and other relevant factors. Two investigators independently extracted the required data from each study, and conflicts were adjudicated by a third author. The quality of the randomized controlled trials was assessed according to the Cochrane risk assessment scale and was determined by evaluating the following factors: random sequence generation method, allocation concealment, blinding, incomplete outcome data, selective outcome reporting, and other sources of bias. We graded each methodological domain as having “low,” “high,” or “unclear” risk of bias. The assessments were performed by two investigators independently. Disagreements were resolved through discussion.

### Data Synthesis and Analysis

We performed a pairwise meta‐analysis for all comparisons listed in the search strategy section by using a random-effects pooling model. Relative risks and standardized mean differences were reported with their 95% CIs. We used the *I* ^2^ statistic to assess heterogeneity among the included trials. A two-sided *p* value of <0.05 was deemed statistically significant. A network meta-analysis was performed to compare the effectiveness of the treatments. First, we summarized the geometry of the network of evidence to compare relationships among treatments. Second, we performed a contrast‐based analysis to compare efficacy. Because of the expected clinical and methodological heterogeneity among the studies in terms of the effects of the treatments, we used a multivariate random-effects model. Treatments were ranked against each other based on their surface under the cumulative ranking (SUCRA) value, with higher SUCRA values representing higher efficacy. Rankograms were used to present a treatment hierarchy for the different drugs.

Inconsistencies among direct and indirect sources of evidence were statistically assessed by a comparing the fit and parsimony of consistency and inconsistency models. The node-splitting method was used to calculate the inconsistency of the model, which separated evidence for a particular comparison into direct and indirect evidence. We tested for small-study effects, such as publication bias using Egger’s test.

## Results

### Literature Search and Study Characteristics

**FIGURE 1:**
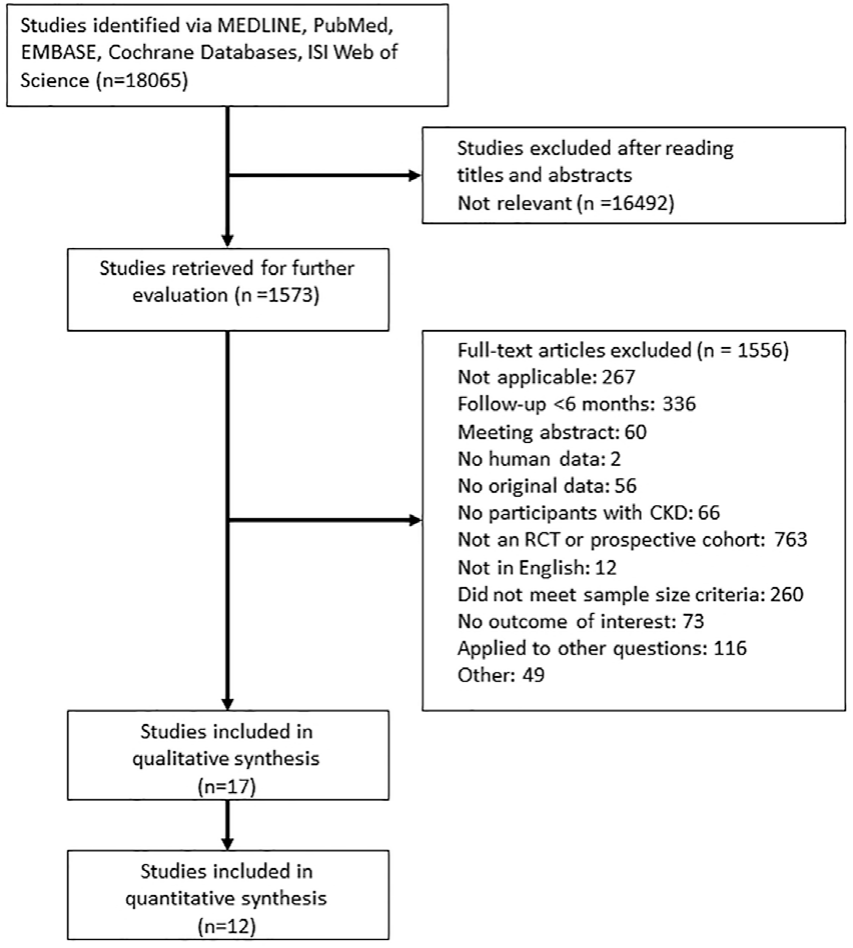
Flowchart for the study review. Abbreviations: CKD, Chronic Kidney Disease; RCT, Randomized controlled trials.

Figure 1 presents a flowchart of the selection process. A total of 18,065 potentially relevant articles were identified after the removal of duplicates. After a review of the abstracts and the full texts, 18,048 of the articles were excluded based on the inclusion criteria, and 17 studies were included (reasons for exclusion are listed in Figure 1). We identified four new studies for this updated review; in addition to the 13 studies that were identified in a previous review (Wilson et al., 2017). Table 1 lists the characteristics of the included studies. The sample size of the study populations ranged from 32 to 4,973, and the follow-up period ranged from 8 to 36 months. Five of the randomized controlled trials included patients diagnosed as having stage 3–5 CKD and patients receiving dialysis (Toussaint et al., 2010; Haghverdi et al., 2014; Shigematsu et al., 2017; Iseri et al., 2019; Sugimoto et al., 2019), and seven studies included patients who had received kidney transplants (Coco et al., 2003; Walsh et al., 2009; Torregrosa et al., 2010; Smerud et al., 2012; Sánchez-Escuredo et al., 2015; Bonani et al., 2016; Marques et al., 2019). Five studies were randomized controlled trials with a subgroup analysis of postmenopausal women with CKD (Jamal et al., 2007; Miller et al., 2007; Ishani et al., 2008; Torregrosa et al., 2010; Jamal et al., 2011). The participants in all of the studies were adults, and the mean age of participants in the study populations ranged from 44 to 80 years.

**TABLE 1:** Characteristics of randomized controlled trials evaluating effects of osteoporosis medications on patients diagnosed as having chronic kidney disease (CKD), receiving dialysis, or having undergone kidney transplantation.

Study, year	Population	Location	Intervention	Comparison	Sample size (*n)*	Follow-up (months)	Selected outcomes	Main reported result(s)	Risk of bias	Funding source
Coco et al. (2003)	Adult men and women post-kidney transplantation	United States	IV pamidronate 60 mg within 48 h after transplantation followed by 30 mg at months 1, 2, 3, and 6	Oral calcitriol and calcium carbonate	59	12	Vertebral and hip BMD, vertebral and hip fractures, renal events, hypocalcemia, hypercalcemia	Pamidronate was more effective in preserving vertebral BMD than treatment in compared groups	Moderate	Not reported
Hernandez et al., 2003 (Jamal et al., 2011)	Postmenopausal women >50 years old receiving dialysis	Venezuela	Oral raloxifene 60 mg daily	Placebo	50	12	Lumbar spine and femoral neck BMD	Raloxifene significantly improved lumbar spine BMD	Moderate	Industry and government
Jamal et al., 2007 (Torregrosa et al., 2010)	Postmenopausal women 55–80 years old, eGFR <45 ml/min	Multicenter, United States	Oral alendronate 5 mg daily	Placebo	581	36 to 48	Lumbar spine, femoral neck, and total hip BMD; clinical fractures and vertebral fractures; GI; CV; cerebrovascular events; cancer; death	Alendronate increased total hip BMD	Moderate	Government
Miller et al., 2007 (Jamal et al., 2007)	Postmenopausal women 42–86 years old, GFR 30–79 ml/min	Multicenter, multicountry	SC teriparatide 20 or 40 mcg daily	Placebo	731	Median, 21	Lumbar spine and femoral neck BMD; vertebral and nonvertebral fractures; renal-related adverse events; hypercalcemia; gout; arthralgia	Teriparatide increased lumbar spine and femoral neck BMD.	Moderate	Industry
								Incidence of vertebral and nonvertebral fractures was lower in patients treated with teriparatide		
Ishani et al., 2008 (Miller et al., 2007)	Postmenopausal women 31–80 years old, CrCl <60 ml/min	Multicenter, multicountry	Oral raloxifene 60 or 120 mg daily	Placebo	4,973	36	Lumbar spine and femoral neck BMD; vertebral and non-vertebral fractures; renal and GI adverse events	Raloxifene treatment was associated with a large increase in spine BMD and a decrease in vertebral fractures	Moderate	Not reported
Walsh et al. (2009)	Post-kidney transplantation men and women 18–75 years old	Multicenter, United Kingdom	IV pamidronate 1 mg/kg at baseline and at 1, 4, 8, and 12 months after transplantation	Control (no bisphosphonates)	93	24	Lumbar spine, femoral neck, total hip, and Ward’s area BMD; fracture rate; hypocalcemia; and renal adverse events	Pamidronate increased the BMD of lumbar spine, total hip, and Ward’s area of the hip	Moderate	Industry
Toussaint et al. (2010)	Men and women 18–80 years old, GFR 20–60 ml/min/1.73 m^2^ and Clcr >25 ml/min	Single-center, Australia	Oral alendronate 70 mg weekly	Placebo	50	18	Lumbar spine and femoral neck BMD; vertebral and hip fracture; GI and renal adverse events; death	Alendronate increased lumbar spine BMD	Moderate	Industry
Torregrosa et al., 2010 (Marques et al., 2019)	Post-kidney transplantation men and women 18–75 years old	Multicenter, Spain	Oral risedronate 35 mg weekly	Oral vitamin D and calcium daily	101	12	Lumbar spine and femoral neck BMD; vertebral fracture; renal adverse events; hypercalcemia; hyperphosphatemia; dyspepsia; death	Risedronate increased lumbar spine BMD at 6- and 12-month follow-up and increased femoral neck BMD only at 6-month follow-up	Moderate	Industry
Jamal et al., 2011 (Ishani et al., 2008)	Postmenopausal women 60–90 years old, stage 3 or 4 CKD	Multicenter, multicountry	SC denosumab 60 mg every 6 months	Placebo	2,890	36	Lumbar spine, femoral neck, and total hip BMD; vertebral fractures; renal, CV, and infection-related adverse events	(Bikbov et al., 2020) Denosumab reduced the incidence of vertebral fractures over 36 months for patients with stage 3 CKD >	Moderate	Industry
								(System URD, 2013) Denosumab increased lumbar spine BMD, femoral neck BMD, and total hip BMD over 36 months for patients with stage 3 CKD		
								(Tentori et al., 2014) Denosumab increased femoral neck BMD and total hip BMD over 36 months for patients with stage 4 CKD		
Smerud et al., 2012 (Torregrosa et al., 2010)	Post-kidney transplantation women and men >18 years old	Single-center, Norway	IV Ibandronate 3 mg every 3 months	Placebo	129	12	Lumbar spine, total femur, ultradistal radius, proximal 1/3 radius, and total body BMD; vertebral fractures; renal adverse events; musculoskeletal pain; infections; death	Ibandronate significantly increased total femur and ultradistal radius BMD	Moderate	Industry
Haghverdi et al. (2014)	Postmenopausal women >40 years old, stage 5 CKD or hemodialysis	Single-center, Iran	Oral raloxifene 60 mg daily	Placebo	51	8	Lumbar spine and femoral neck BMD; vertebral fractures	Raloxifene significantly increased lumbar spine BMD	Moderate	Not reported
Sánchez-Escuredo et al., 2015 (Smerud et al., 2012)	Post-kidney transplantation women and men 50–75 years old	Single-center, Spain	Oral ibandronate 150 mg monthly	Oral risedronate 35 mg weekly	69	12	Lumbar spine and femoral neck BMD; GI and renal adverse events; death	Both monthly oral ibandronate and weekly oral risedronate increased lumbar spine BMD	Moderate	Not reported
Bonani et al., 2016 (17)	Post-kidney transplantation adult men and women	Single-center, Switzerland	SC denosumab 60 mg every 6 months	Control (no treatment)	90	12	Lumbar spine, femoral neck, and total hip BMD; fracture; renal and GI adverse events; musculoskeletal pain; hypocalcemia; hypercalcemia; infections; death	Denosumab increased total lumbar spine and total hip areal BMD	Moderate	University and university hospital
Shigematsu et al., 2017 (Torregrosa et al., 2010)	Men and women >40 years old with stage 3 CKD	Multicenter, Japan	Oral risedronate 2.5 mg once daily	Oral risedronate 17.5 mg once weekly or intermittent oral etidronate (one cycle: 2 weeks of 200 mg once daily followed by 10 weeks off)	228	12–24	Lumbar spine BMD, atypical femoral fractures, renal and GI adverse events, hypocalcemia, hypercalcemia, osteonecrosis of the jaw	Risedronate increased lumbar spine BMD	Moderate	Industry
Iseri et al., 2019 (Iseri et al., 2019)	Men and women >20 years old undergoing hemodialysis	Multicenter, Japan	SC denosumab 60 mg every 6 months	IV alendronate 900 mg every 4 weeks	46	12	Lumbar spine, femoral neck, and distal radius BMD; vertebral fracture; GI adverse events; musculoskeletal pain; hypocalcemia; hypercalcemia; infections; death	Denosumab and alendronate both significantly increased lumbar spine BMD	Moderate	Industry
Marques et al., 2019 (Bonani et al., 2016)	Post-kidney transplantation, ≥18 years old	Single-center, Brazil	IV zoledronate 5 mg once	Control (cholecalciferol)	32	12	Lumbar spine, femoral neck, and total hip BMD; renal adverse events; hypocalcemia; hypercalcemia	Zoledronate increased lumbar spine and total hip BMD	Moderate	Government
Sugimoto et al., 2019 (Sugimoto et al., 2019)	CKD stage G3 (eGFR ≥30 to <60 ml/min/1.73 m^2^), men >50 years old and women >50 years old ≧2 years after menopause	Multicenter, Japan	Oral risedronate 75 mg monthly	Oral calcium lactate daily	41	12	Lumbar spine BMD, incidence of adverse events	Lumbar spine BMD significantly increased from baseline at months 6 and 12	Moderate	Industry

Abbreviations: CKD, chronic kidney disease; BMD, bone mineral density; IV, intravenous; SC, subcutaneous; CV, cardiovascular; GI, gastrointestinal.

### Risk of Bias

**FIGURE 2:**
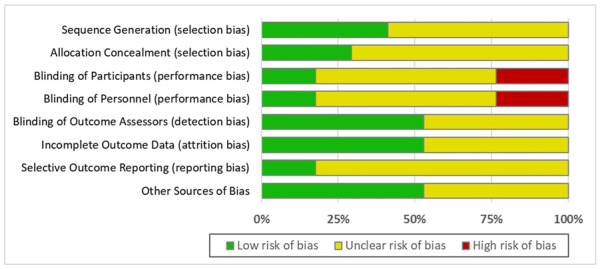
Risk of bias assessment.

The included studies exhibited moderate to high risk of bias (Figure 2). The majority of the studies did not report an adequate amount of information regarding sequence generation or allocation concealment or had selective outcome reporting. Only three studies described blinding of participants and study personnel (Hernández et al., 2003; Torregrosa et al., 2010; Sugimoto et al., 2019). Nine studies described blinding of outcome assessors (Coco et al., 2003; Hernández et al., 2003; Walsh et al., 2009; Torregrosa et al., 2010; Toussaint et al., 2010; Iseri et al., 2019). The majority of the studies either received industry funding (Hernández et al., 2003; Jamal et al., 2007; Ishani et al., 2008; Walsh et al., 2009; Torregrosa et al., 2010; Toussaint et al., 2010; Jamal et al., 2011; Iseri et al., 2019; Sugimoto et al., 2019) or did not report their funding source (Coco et al., 2003; Miller et al., 2007; Smerud et al., 2012; Haghverdi et al., 2014). Three studies were funded by nonprofit or government organizations (Torregrosa et al., 2010; Sánchez-Escuredo et al., 2015; Bonani et al., 2016).

### Vertebral and Clinical Fractures

**FIGURE 3:**
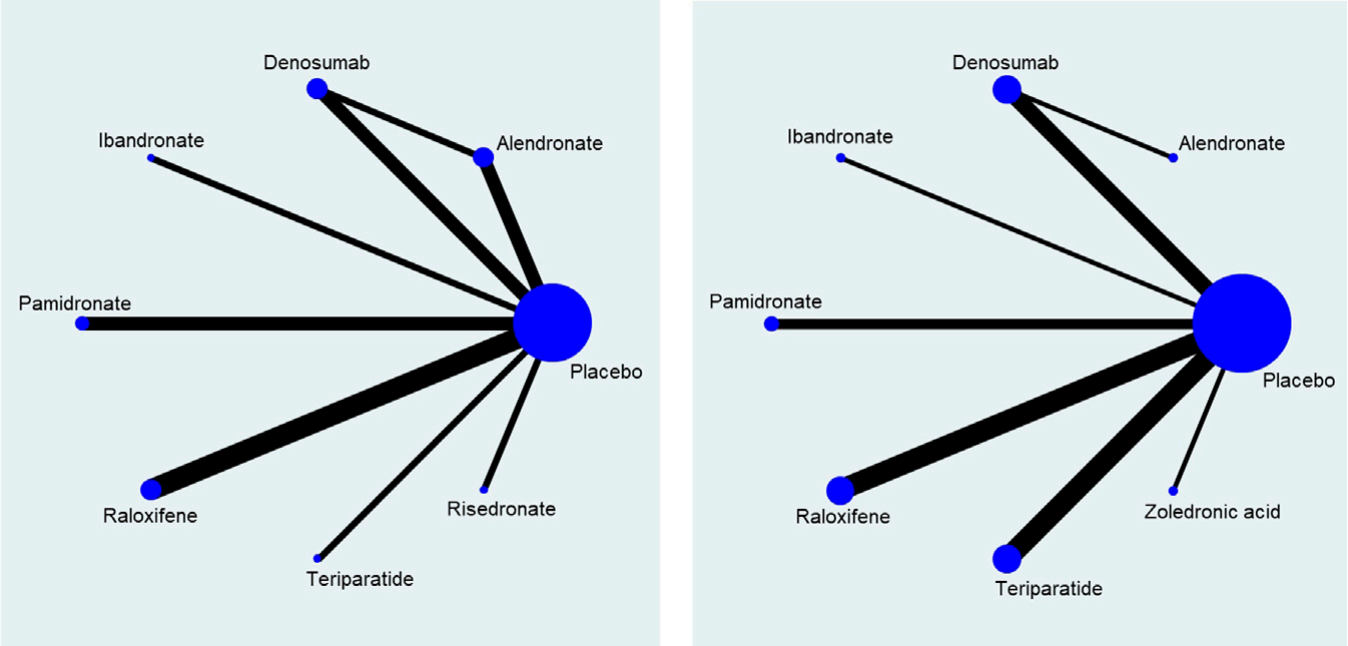
Network of direct comparison for the treatment of Osteoporosis. Each node represents one treatment. The size of the node is proportional to the number of participants randomized to that treatment. The edges represent direct comparisons, and the width of the edge is proportional to the number of trials. **(A)** map for risk of fracture; **(B)** map for percentage change of bone mineral density.

**FIGURE 4:**
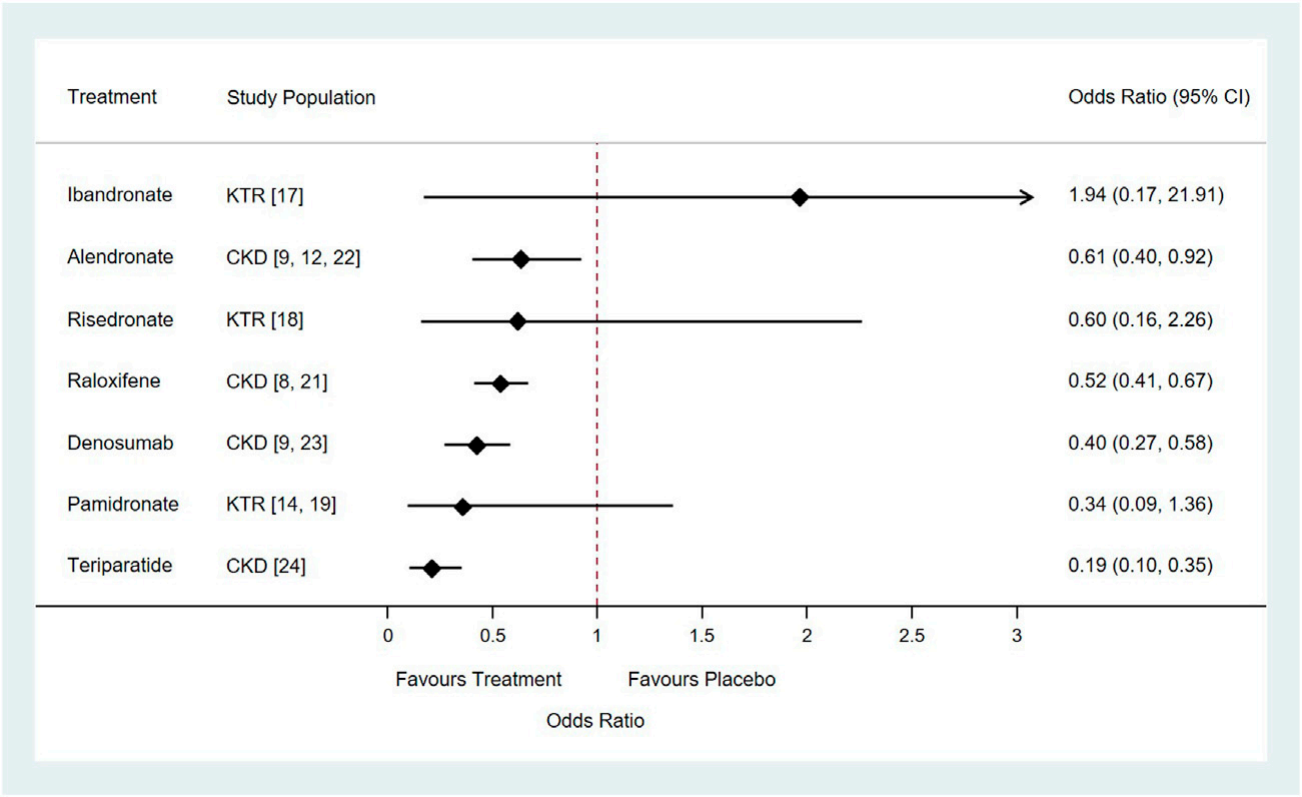
Forest plots of relative risk of vertebral or clinical fractures of seven drugs for the treatment of osteoporosis compared with placebo in patients with chronic kidney disease or underwent kidney transplantation. Abbreviation: CKD, Chronic Kidney Disease; KTR, Kidney Transplant Recipients.

A total of eight different medications and placebo control were included in the network meta-analysis: the direct comparisons made for different outcomes are shown in Figure 3. Results of the pairwise meta-analyses and related heterogeneity are reported in the appendix (Supplementary Figures S2–S4). With respect to new vertebral or clinical fracture events, treatment with alendronate, denosumab, raloxifene, or teriparatide were all associated with a significantly lower risk of new vertebral or clinical fractures compared to treatment with placebos [alendronate: odds ratio (OR) = 0.61, 95% CI: 0.40–0.92; raloxifene: OR = 0.52, 95% CI: 0.41–0.67; denosumab: OR = 0.40, 95% CI: 0.27–0.58; teriparatide: OR = 0.19, 95% CI: 0.10–0.35]. Treatment with two other medications, pamidronate and risedronate, was also had associated with vertebral fracture, however the association did not reach statistical significance (pamidronate: OR = 0.34, 95% CI: 0.09–1.36; risedronate: OR = 0.60, 95% CI: 0.16–2.26) (Figure 4). It is worth to notice, the data of vertebral or clinical fractures on pamidronate, ibandronate, and risedronate were only reported for the study population of kidney transplant patients, thus these results should be interpreted cautiously.

### BMD

**FIGURE 5:**
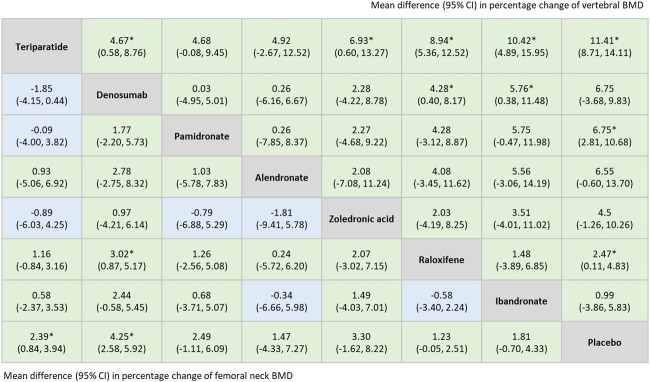
Summary estimates, (mean difference with 95% CI), for percentage change of vertebral BMD and femoral neck BMD derived from network meta-analysis of 11 trials. Results of percentage change of vertebral BMD were listed in the upper triangle, [the comparison is row vs. column (comparator)], and the results of percentage change of femoral neck BMD were listed in lower triangle [the comparison is column vs. row (comparator)]. *: p < 0.05. Abbreviations: BMD, Bone Mineral Density.

The network meta-analysis indicated that among all included treatments, teriparatide and denosumab were superior options for improving BMD at the lumbar spine and the femoral neck. Treatment with teriparatide, pamidronate, or raloxifene were all significantly associated with an increase in vertebral BMD compared to treatment with a placebo [weighted mean difference (WMD) = 11.41, 95% CI = 8.71–14.11; WMD = 6.75, 95% CI = 2.81–10.68; WMD = 2.47, 95% CI = 0.11–4.83, respectively]. Patients treated with teriparatide or denosumab exhibited significant improvements in femoral neck BMD compared with those treated with placebos (WMD = 2.39, 95% CI = 0.84–3.94; WMD = 4.25, 95% CI = 2.58–5.92, respectively) (Figure 5).

### Cumulative Probability of Treatment Efficacy

**FIGURE 6:**
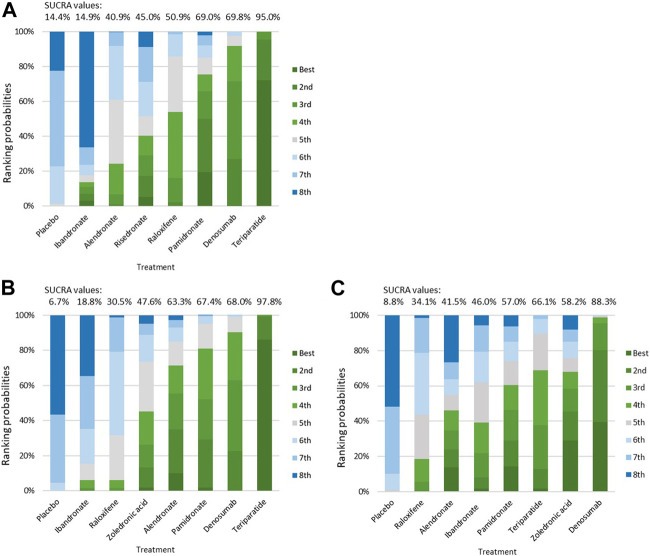
Ranking probabilities for the treatments for Osteoporosis in patients withchronic kidney disease. **(A)** histogram for risk of fracture; **(B)** histogram for percentage change of vertebral BMD; **(C)** histogram for percentage change of femoral neck BMD. Abbreviation: BMD, Bone Mineral Density.

According to the rankogram for risk of vertebral or clinical fracture, teriparatide was the best treatment, with a SUCRA value of 95.0%, followed by denosumab (69.8%), pamidronate (69.0%), raloxifene (50.9%), risedronate (45.0%), alendronate (40.9%), ibandronate (14.9%), and the placebo (14.4%; Figure 6A). The results in Figures 6B,C indicate that teriparatide achieved the highest SUCRA value for change in BMD at the lumbar spine (97.8%), whereas denosumab exhibited the highest SUCRA value for change in BMD at the femoral neck (88.3%). Thus, teriparatide and denosumab had relatively high efficacy as treatments for osteoporosis in patients with CKD, patients receiving dialysis, or patients with a history of kidney transplantation.

### Adverse Events

Supplementary Table S1 summarizes the adverse effects reported in the studies. Seven studies reported death, with mortality rate ranging from 0 to 11.05%. No study reported a significant difference in mortality rate between the treatment and control groups. Four studies reported the incidence of infection such as urinary tract infection, pneumonia and viremia. Bonani et al. reported a higher incidence of urinary tract infection in the denosumab group than in the control group (*p* = 0.008) (Sánchez-Escuredo et al., 2015). However, another trial reported no statistically significant difference in serious infection between denosumab and placebo groups (Ishani et al., 2008).

Twelve studies reported renal adverse events. One trial reported a small but statistically significant difference (*p* = 0.02) in the change in serum creatinine levels from baseline to the third year between the denosumab group (−1.53 mmol/L) and the placebo group (−3.1 mmol/L) for patients with stage 3 or 4 CKD (Ishani et al., 2008). Eleven other trials found that changes in serum creatinine levels, the number of renal-related adverse events, and the rates of kidney transplantation rejection were similar among the treatment and control groups.

Eight studies reported gastrointestinal adverse events. Only one study (Sánchez-Escuredo et al., 2015) reported a larger number of diarrhea events, with a statistically significant difference between the denosumab group (50.0%) and the control group (29.5%). Thirteen trials reported changes in calcium levels. Ten studies documented hypocalcemia events. Two studies reported a significantly higher incidence of transient hypocalcemia in the denosumab group vs. the control or alendronate group (26.1 vs. 2.3%, and 27.3 vs. 4.2%). Twelve studies reported the incidence of hypercalcemia. Miller et al. (Jamal et al., 2007) observed that the incidence of hypercalcemia was higher 4–6 h postdose for patients treated with teriparatide than for those receiving the placebo. In addition, a significantly higher incidence of hypercalcemia was observed in the alendronate group (37.5%) within the first 2 weeks of treatment than in the denosumab group (9.1%) (Iseri et al., 2019).

For serious cardiovascular events, musculoskeletal pain, and the occurrence of hypophosphatemia or hyperphosphatemia, no statistically significant difference was observed between the treatment and control groups in any of the included studies. None of these studies reported hypersensitivity reactions or osteonecrosis of the jaw.

## Discussion

This meta-analysis was performed on 17 randomized controlled trials comprising 10,214 patients who were diagnosed as having stage 2–5 CKD, were receiving dialysis, or had undergone kidney transplantation. Our study found that teriparatide and denosumab exhibited a relatively high efficacy in reducing the risk of fracture and improving vertebral and femoral neck BMD. Teriparatide ranked first in terms of lowering the risk of fracture and improving vertebral BMD, and denosumab ranked second. Denosumab ranked first in terms of improving femoral neck BMD, and teriparatide ranked second. On the other hand, ibandronate, pamidronate, and risedronate were not found to be effective at reducing the risk of vertebral or clinical fracture in patients with CKD. No major or severe safety issue was found with osteoporosis medications from our study. We consider other minor adverse events may be acceptable and treatable for patients with CKD.

Treatment for osteoporosis among patients with CKD consists of antiresorptive agents and anabolic agents (Shigematsu et al., 2017). In general, antiresorptive agents are used to treat patients with normal-to high-turnover bone disease, whereas anabolic agents are used for low-turnover bone disease (Shigematsu et al., 2017; Hsu et al., 2020). Although bone biopsy is the gold standard for diagnosis of renal osteodystrophy, its use is limited by its invasiveness and lack of availability in most hospitals. In addition, the bone component of CKD-associated osteodystrophy can be mixed or vary with time (Khairallah and Nickolas, 2018). Therefore, the efficacy of osteoporosis medications for patients with CKD remains unknown. According to the results of our analysis, teriparatide, (an anabolic agent), and denosumab, (an antiresorptive agent), are superior options for preventing fracture and increasing BMD in patients with CKD.

Teriparatide is a recombinant peptide of the first 34 amino-N-terminal residues of parathormone (PTH), which is given daily via subcutaneous route for treatment of age-related and glucocorticoid-induced osteoporosis (Drüeke and Massy, 2016). Denosumab is a fully human monoclonal antibody to the receptor activator of nuclear factor-κB ligand. By inhibiting the development and the activity of osteoclasts, denosumab reduces bone resorption and increases BMD (Neer et al., 2001). Denosumab is given once every 6 months subcutaneously and should be injected by health care professionals. Compared to teriparatide and other antiresorptive agents, denosumab has a lower dosing frequency and therefore appears to have better persistence and adherence. Denosumab is administered every 6 months and is not eliminated by the kidneys; therefore, denosumab may be easier to use and be less likely cause safety issues in other organ systems than the other antiresorptive agents.

According to our meta-analysis, ibandronate, pamidronate, and risedronate were not effective at reducing the risk of fracture in patients with CKD or patients with a history of kidney transplantation. The relative risk of fracture for patients receiving ibandronate was higher than for patients receiving the placebo. Of the two clinical trials evaluating the efficacy of ibandronate that were included in our meta-analysis (Torregrosa et al., 2010; Smerud et al., 2012), only one trial evaluated the risk of fracture (Torregrosa et al., 2010). Smerud reported that among 129 recipients of kidney transplants, vertebral fracture was observed in two patients in the ibandronate group, (total 66 patients), and one in the placebo group (total 63 patients) (Torregrosa et al., 2010). However, their findings may have been limited by the small sample size, the low number of events, and the patient groups being limited to recipients of kidney transplants.

Other meta-analyses comparing the effects of osteoporosis medications on patients with CKD and patients with a history of kidney transplantation with that of placebos (Delmas, 2008; Wilson et al., 2017) have shown that various antiresorptive agents and anabolic agents exhibit relatively high efficacy in improving BMD and reducing the risk of fracture. Although studies have examined the effects of various drugs during the treatment of osteoporosis on patients with CKD, no consensus has been reached regarding the best drug.

Our study also investigated the mortality rate and adverse events associated with the osteoporosis medications. We found no severe safety issue but minor and acceptable adverse events for patients with CKD in this study. Nevertheless, our review of the safety of osteoporosis medications is not comprehensive because the studies included in our analysis may have selectively reported outcomes and had a limited duration of follow-up. Furthermore, hypocalcemia was reported in two of the studies (Sánchez-Escuredo et al., 2015; Iseri et al., 2019) that used denosumab in the treatment group. Although the population characteristics and the small number of patients in the two trials limited the usefulness of their results in our network meta-analysis, both studies reported the adverse event of hypocalcemia in patients with CKD and a history of kidney transplantation following treatment with denosumab (Kan et al., 2016). Caution should be exercised when treating osteoporosis in patients with CKD and a history of kidney transplantation with denosumab.

Compared with previous meta-analyses, our study has several strengths. First, we performed a network meta-analysis to synthesize both direct and indirect evidence, which can aid clinicians in decision-making. Second, our review included patients with stage 2–5 CKD, whereas the majority of relevant meta-analyses were limited to an analysis of kidney transplantation recipients. Our study also has several limitations. First, osteodystrophy associated with CKD can vary in type over time (Khairallah and Nickolas, 2018). Normal-to high-turnover bone disease is common in early stage CKD, whereas low-turnover bone disease is common in end-stage CKD as kidney function declines. Thus, the severity of kidney disease in the studies included in our meta-analysis could have contributed to the heterogeneity of the results. Second, the findings from our meta-analysis may have limited generalizability because of the specific subgroupings used in some of the included studies. Among the 17 studies, five studies included only postmenopausal women, and seven studies were limited to recipients of kidney transplants. Teriparatide was investigated in only a single study for post-menopausal women with CKD stage 2–3. Denosumab was investigated in three different trials, conducted among patients with CKD stage 3–5, hemodialysis, and renal transplantation. Data on Pamidronate and Ibandronate were limited to renal transplant patients, while Alendronate was tested only among CKD patients with or without hemodialysis. Risedronate was investigated among patients with CKD and patients who had received a kidney transplant but not in dialysis patients. Also, since the data of vertebral or clinical fractures on Pamidronate, Ibandronate, and Risedronate were only reported for the study population of kidney transplant patients, the comparison should be interpreted cautiously. Third, several of the participants in the trials may have received cointerventions, such as calcium or vitamin D supplements, which may have influenced the results. Fourth, in this meta-analysis we did not consider the potential effects of baseline circulating levels of parathormone (PTH), bone specific alkaline phosphatase (BSAP), calcium (Ca) and phosphate (P), which may influence the comparison between osteoporosis treatments and placebo controls, especially when the sample size of the study was small. Fifth, the length of follow-up varied among the studies, resulting in potentially significant variations in the incidence of adverse effects. Finally, the majority of the studies included in our meta-analysis exhibited moderate to high risk of bias because their methods were not clearly explained.

For patients with CKD or a history of kidney transplantation, teriparatide seems to be the most effective treatment for preventing new vertebral or clinical fractures, while denosumab displays the greatest improvement in femoral neck BMD. However, the possibility of hypocalcemia occurring should be considered when denosumab is used as the treatment. Although no significant difference in safety outcomes was observed between the osteoporosis medications and placebo controls, clinicians should consider the balance of benefits and harms, values and preferences, and cost, for the best therapeutic options for CKD patients with osteoporosis. Because of the limitations and potential bias in the studies included in our meta-analysis, our findings should be interpreted with caution. Additional randomized control trials with high-quality data, sufficient follow-up times, and examination of distinct subgroups based on CKD stage are required. While this study this study should be useful to clinicians because it synthesizes existing evidence, the best options for treatment of osteoporosis in patients with CKD remains undetermined.

## Data Availability

The original contributions presented in the study are included in the article/Supplementary Material, further inquiries can be directed to the corresponding author.

## References

[B1] Bikbov B. Purcell C. A. Levey A. S. Smith M. Abdoli A. Abebe M. (2020). Global, Regional, and National burden of Chronic Kidney Disease, 1990-2017: a Systematic Analysis for the Global Burden of Disease Study 2017. Lancet 395, 709–733. 10.1016/S0140-6736(20)30045-3 32061315 PMC7049905

[B2] Bonani M. Frey D. Brockmann J. Fehr T. Mueller T. F. Saleh L. (2016). Effect of Twice-Yearly Denosumab on Prevention of Bone Mineral Density Loss in De Novo Kidney Transplant Recipients: A Randomized Controlled Trial. Am. J. Transpl. 16, 1882–1891. 10.1111/ajt.13692 26713403

[B3] Coco M. Glicklich D. Faugere M. C. Burris L. Bognar I. Durkin P. (2003). Prevention of Bone Loss in Renal Transplant Recipients: a Prospective, Randomized Trial of Intravenous Pamidronate. J. Am. Soc. Nephrol. 14, 2669–2676. 10.1097/01.asn.0000087092.53894.80 14514747

[B4] Delmas P. D. (2008). Clinical Potential of RANKL Inhibition for the Management of Postmenopausal Osteoporosis and Other Metabolic Bone Diseases. J. Clin. Densitom. 11, 325–338. 10.1016/j.jocd.2008.02.002 18375161

[B5] Drüeke T. B. Massy Z. A. (2016). Changing Bone Patterns with Progression of Chronic Kidney Disease. Kidney Int. 89, 289–302. 10.1016/j.kint.2015.12.004 26806832

[B6] Group KDIGOC-MW (2009). KDIGO Clinical Practice Guideline for the Diagnosis, Evaluation, Prevention, and Treatment of Chronic Kidney Disease-Mineral and Bone Disorder (CKD-MBD). Kidney Int. Suppl. 113, S1–S130. 10.1038/ki.2009.188 19644521

[B7] Haghverdi F. Farbodara T. Mortaji S. Soltani P. Saidi N. (2014). Effect of Raloxifene on Parathyroid Hormone in Osteopenic and Osteoporotic Postmenopausal Women with Chronic Kidney Disease Stage 5. Iran J. Kidney Dis. 8, 461–466. 25362221

[B8] Hernández E. Valera R. Alonzo E. Bajares-Lilue M. Carlini R. Capriles F. (2003). Effects of Raloxifene on Bone Metabolism and Serum Lipids in Postmenopausal Women on Chronic Hemodialysis. Kidney Int. 63, 2269–2274. 10.1046/j.1523-1755.2003.00005.x 12753317

[B9] Hsu C.-Y. Chen L.-R. Chen K.-H. (2020). Osteoporosis in Patients with Chronic Kidney Diseases: A Systemic Review. Ijms 21, 6846. 10.3390/ijms21186846 PMC7555655 32961953

[B10] Iseri K. Watanabe M. Yoshikawa H. Mitsui H. Endo T. Yamamoto Y. (2019). Effects of Denosumab and Alendronate on Bone Health and Vascular Function in Hemodialysis Patients: a Randomized, Controlled Trial. J. Bone Miner Res. 34, 1014–1024. 10.1002/jbmr.3676 30690785

[B11] Ishani A. Blackwell T. Jamal S. A. Cummings S. R. Ensrud K. E. (2008). The Effect of Raloxifene Treatment in Postmenopausal Women with CKD. J. Am. Soc. Nephrol. 19, 1430–1438. 10.1681/ASN.2007050555 18400939 PMC2440292

[B12] Jamal S. A. Bauer D. C. Ensrud K. E. Cauley J. A. Hochberg M. Ishani A. (2007). Alendronate Treatment in Women with normal to Severely Impaired Renal Function: an Analysis of the Fracture Intervention Trial. J. Bone Miner Res. 22, 503–508. 10.1359/jbmr.070112 17243862

[B13] Jamal S. A. Ljunggren O. Stehman-Breen C. Cummings S. R. McClung M. R. Goemaere S. (2011). Effects of Denosumab on Fracture and Bone mineral Density by Level of Kidney Function. J. Bone Miner Res. 26, 1829–1835. 10.1002/jbmr.403 21491487

[B14] Kan S. L. Ning G. Z. Chen L. X. Zhou Y. Sun J. C. Feng S. Q. (2016). Efficacy and Safety of Bisphosphonates for Low Bone mineral Density after Kidney Transplantation: a Meta-Analysis. Medicine (Baltimore) 95, e2679. 10.1097/MD.0000000000002679 26844505 PMC4748922

[B15] Ketteler M. Block G. A. Evenepoel P. Fukagawa M. Herzog C. A. McCann L. (2017). Executive Summary of the 2017 KDIGO Chronic Kidney Disease-Mineral and Bone Disorder (CKD-MBD) Guideline Update: What's Changed and Why it Matters. Kidney Int. 92, 26–36. 10.1016/j.kint.2017.04.006 28646995

[B16] Khairallah P. Nickolas T. L. (2018). Management of Osteoporosis in CKD. Clin. J. Am. Soc. Nephrol. 13, 962–969. 10.2215/CJN.11031017 29487093 PMC5989687

[B17] Marques I. D. B. Araújo M. J. C. L. N. Graciolli F. G. Dos Reis L. M. Pereira R. M. R. Alvarenga J. C. (2019). A Randomized Trial of Zoledronic Acid to Prevent Bone Loss in the First Year after Kidney Transplantation. J. Am. Soc. Nephrol. 30, 355–365. 10.1681/ASN.2018060656 30606784 PMC6362629

[B18] Miller P. D. Schwartz E. N. Chen P. Misurski D. A. Krege J. H. (2007). Teriparatide in Postmenopausal Women with Osteoporosis and Mild or Moderate Renal Impairment. Osteoporos. Int. 18, 59–68. 10.1007/s00198-006-0189-8 17013567

[B19] Neer R. M. Arnaud C. D. Zanchetta J. R. Prince R. Gaich G. A. Reginster J. Y. (2001). Effect of Parathyroid Hormone (1-34) on Fractures and Bone mineral Density in Postmenopausal Women with Osteoporosis. N. Engl. J. Med. 344, 1434–1441. 10.1056/NEJM200105103441904 11346808

[B20] Pisani P. Renna M. D. Conversano F. Casciaro E. Di Paola M. Quarta E. (2016). Major Osteoporotic Fragility Fractures: Risk Factor Updates and Societal Impact. World J. Orthop. 7, 171–181. 10.5312/wjo.v7.i3.171 27004165 PMC4794536

[B21] Sánchez-Escuredo A. Fuster D. Rubello D. Muxí A. Ramos A. Campos F. (2015). Monthly Ibandronate versus Weekly Risedronate Treatment for Low Bone mineral Density in Stable Renal Transplant Patients. Nucl. Med. Commun. 36, 815–818. 10.1097/MNM.0000000000000316 25856225

[B22] Shigematsu T. Muraoka R. Sugimoto T. Nishizawa Y. (2017). Risedronate Therapy in Patients with Mild-To-Moderate Chronic Kidney Disease with Osteoporosis: post-hoc Analysis of Data from the Risedronate Phase III Clinical Trials. BMC Nephrol. 18, 66. 10.1186/s12882-017-0478-9 28201994 PMC5311729

[B23] Smerud K. T. Dolgos S. Olsen I. C. Åsberg A. Sagedal S. Reisæter A. V. (2012). A 1-year Randomized, Double-Blind, Placebo-Controlled Study of Intravenous Ibandronate on Bone Loss Following Renal Transplantation. Am. J. Transpl. 12, 3316–3325. 10.1111/j.1600-6143.2012.04233.x 22946930

[B24] Sugimoto T. Inoue D. Maehara M. Oikawa I. Shigematsu T. Nishizawa Y. (2019). Efficacy and Safety of Once-Monthly Risedronate in Osteoporosis Subjects with Mild-To-Moderate Chronic Kidney Disease: a Post Hoc Subgroup Analysis of a Phase III Trial in Japan. J. Bone Miner Metab. 37, 730–740. 10.1007/s00774-018-0977-1 30523414

[B25] System URD (2013). USRDS 2013 Annual Data Report: Atlas of Chronic Kidney Disease and End-Stage Renal Disease in the United States, Bethesda, MD, USA: National Institutes of Health, National Institute of Diabetes and Digestive and Digestive and Kidney Diseases.

[B26] Tentori F. McCullough K. Kilpatrick R. D. Bradbury B. D. Robinson B. M. Kerr P. G. (2014). Response to High Rates of Death and Hospitalization Follow Bone Fracture Among Hemodialysis Patients. Kidney Int. 85, 166–173. 10.1038/ki.2013.279 25152548 PMC4141532

[B29] Torregrosa J. V. Fuster D. Gentil M. A. Marcen R. Guirado L. Zarraga S. (2010). Open-label Trial: Effect of Weekly Risedronate Immediately after Transplantation in Kidney Recipients. Transplantation 89, 1476–1481. 10.1097/TP.0b013e3181dc13d0 20393402

[B31] Toussaint N. D. Lau K. K. Strauss B. J. Polkinghorne K. R. Kerr P. G. (2010). Effect of Alendronate on Vascular Calcification in CKD Stages 3 and 4: a Pilot Randomized Controlled Trial. Am. J. Kidney Dis. 56, 57–68. 10.1053/j.ajkd.2009.12.039 20347511

[B32] Walsh S. B. Altmann P. Pattison J. Wilkie M. Yaqoob M. M. Dudley C. (2009). Effect of Pamidronate on Bone Loss after Kidney Transplantation: a Randomized Trial. Am. J. Kidney Dis. 53, 856–865. 10.1053/j.ajkd.2008.11.036 19393473

[B33] Wilson L. M. Rebholz C. M. Jirru E. Liu M. C. Zhang A. Gayleard J. (2017). Benefits and Harms of Osteoporosis Medications in Patients with Chronic Kidney Disease: a Systematic Review and Meta-Analysis. Ann. Intern. Med. 166, 649–658. 10.7326/M16-2752 28395318

