# Supplementary material for: Efficacy of Osteoporosis Medications for Patients With Chronic Kidney Disease: An Updated Systematic Review and Network Meta-Analysis
Source: Front Pharmacol. 2022 Feb 11;13:822178. doi: 10.3389/fphar.2022.822178 (PMC8873386; doi:10.3389/fphar.2022.822178)
Supplement: Supplementary file 1 [file DataSheet1.docx]

Supplementary Material

# Search Strategy

1. "Kidney Diseases"[Mesh] OR "Renal Dialysis"[Mesh] OR renal[tiab] OR kidney[tiab] OR nephro*[tiab] OR hemodialysis[tiab] OR haemodialysis[tiab]
2. Biopsy[Mesh] OR "Bone Density"[Mesh] OR "bone mineral density"[tiab] OR "x-ray absorptiometry"[tiab] OR DXA[tiab]
3. Alendronate[Mesh] OR "Etidronic Acid"[Mesh] OR Teriparatide[Mesh] OR Raloxifene[Mesh] OR bisphosphonate*[tiab] OR pamidronate[tiab] OR alendronate[tiab] OR teriparatide[tiab] OR raloxifene[tiab] OR denosumab[tiab] OR risedronate[tiab]
4. Calcium/blood[Mesh] OR Parathyroid Hormone[Mesh] OR Phosphorus/blood[Mesh] OR (serum[tiab] AND (phosphorus[tiab] OR calcium[tiab] OR PTH[tiab] OR "parathyroid hormone"[tiab] OR phosphate[tiab]))
5. "Calcium Carbonate"[Mesh] Or "Calcium Citrate"[Mesh] OR Lanthanum[Mesh] OR "aluminum hydroxide"[tiab] OR "calcium acetate"[tiab] OR "calcium carbonate"[tiab] OR "calcium citrate"[tiab] OR "calcium ketoglutarate"[tiab] OR "calcium gluconate"[tiab] OR "ferric citrate"[tiab] OR "magnesium/calcium carbonate"[tiab] OR " calcium acetate/magnesium carbonate "[tiab] OR sevelamer[tiab] OR lanthanum[tiab] OR "sucroferric oxyhydroxide"[tiab] OR ((phosphorus* OR phosphate*[tiab]) AND (binder*[tiab] OR binding*[tiab]))
6. "Phosphorus, dietary"[Mesh] OR (diet*[tiab] AND (phosphate[tiab] OR phosphorus[tiab]) AND (restrict*[tiab] OR limit*[tiab]))
7. "Vitamin D"[Mesh] OR "vitamin D"[tiab] OR cholecalciferol*[tiab] OR ergocalciferol*[tiab] OR ercalcidiol*[tiab] OR calcidiol*[tiab] OR calcitriol*[tiab] OR doxercalciferol*[tiab] OR paricalcitol*[tiab] OR alfacalcidol*[tiab] OR falecalcitriol*[tiab] OR maxacalcitol*[tiab]
8. #1 AND #2
9. #1 AND #3
10. #1 AND #4
11. #1 AND #5
12. #1 AND #6
13. #1 AND #7
14. #8 OR #9 OR #10 OR #11 OR #12 OR #13
15. #14 AND ("systematic review"[ti] OR "meta-analysis"[ti])
16. #14 NOT #15
17. #16 AND Eng[la] NOT (animal[mh] NOT human[mh]) Filters: from 2017 - 2020

# Supplementary Figures and Tables

## Supplementary Figures


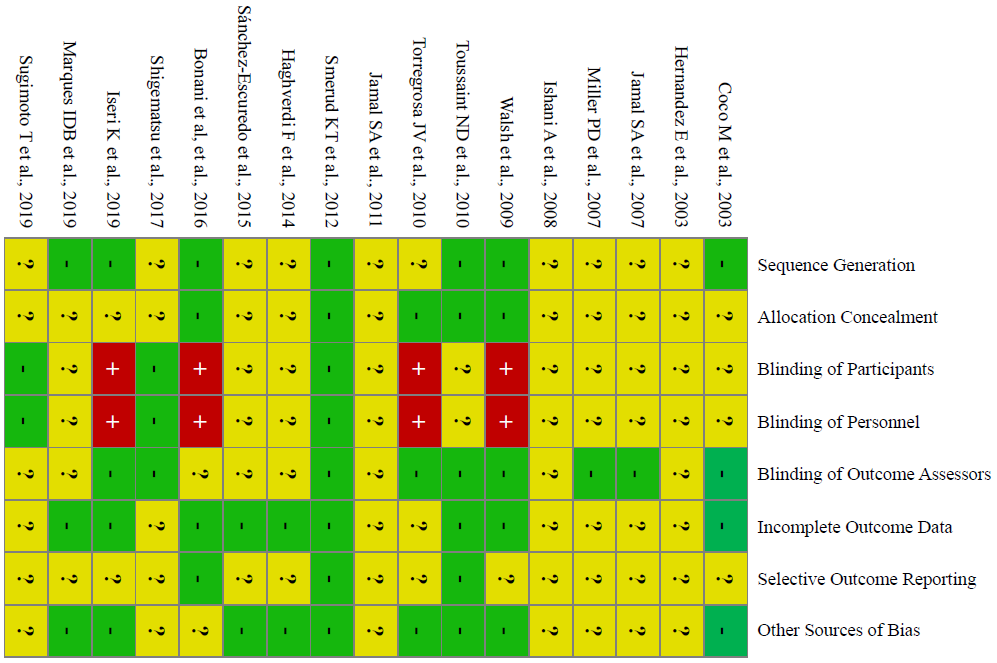


**Supplementary Figure 1.** Assessment of risk of bias in the included trials

**
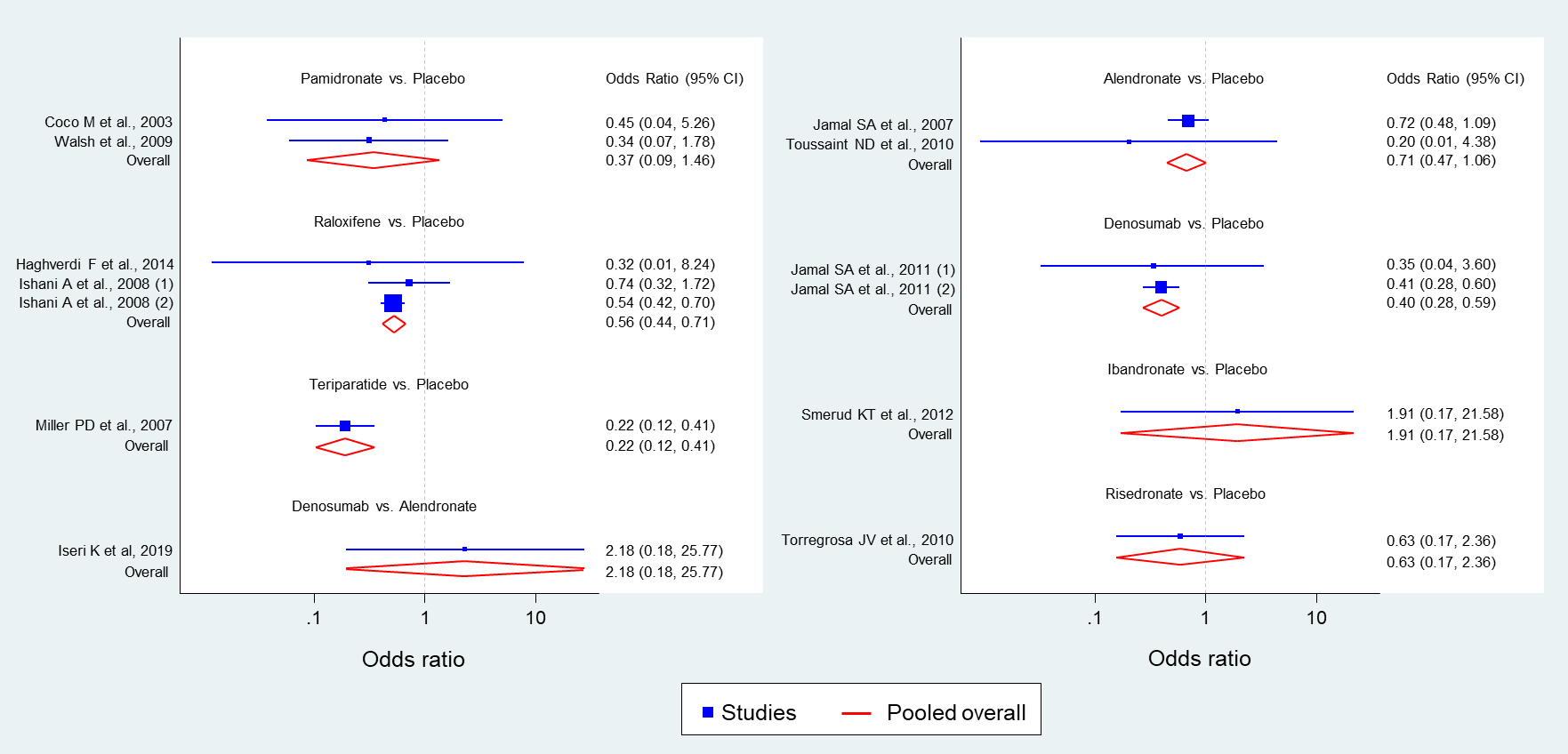
**

**Supplementary Figure 2.** Summary of the head-to-head comparisons of relative risk of vertebral or clinical fractures for the treatment of osteoporosis in patients with CKD or underwent kidney transplantation.

**
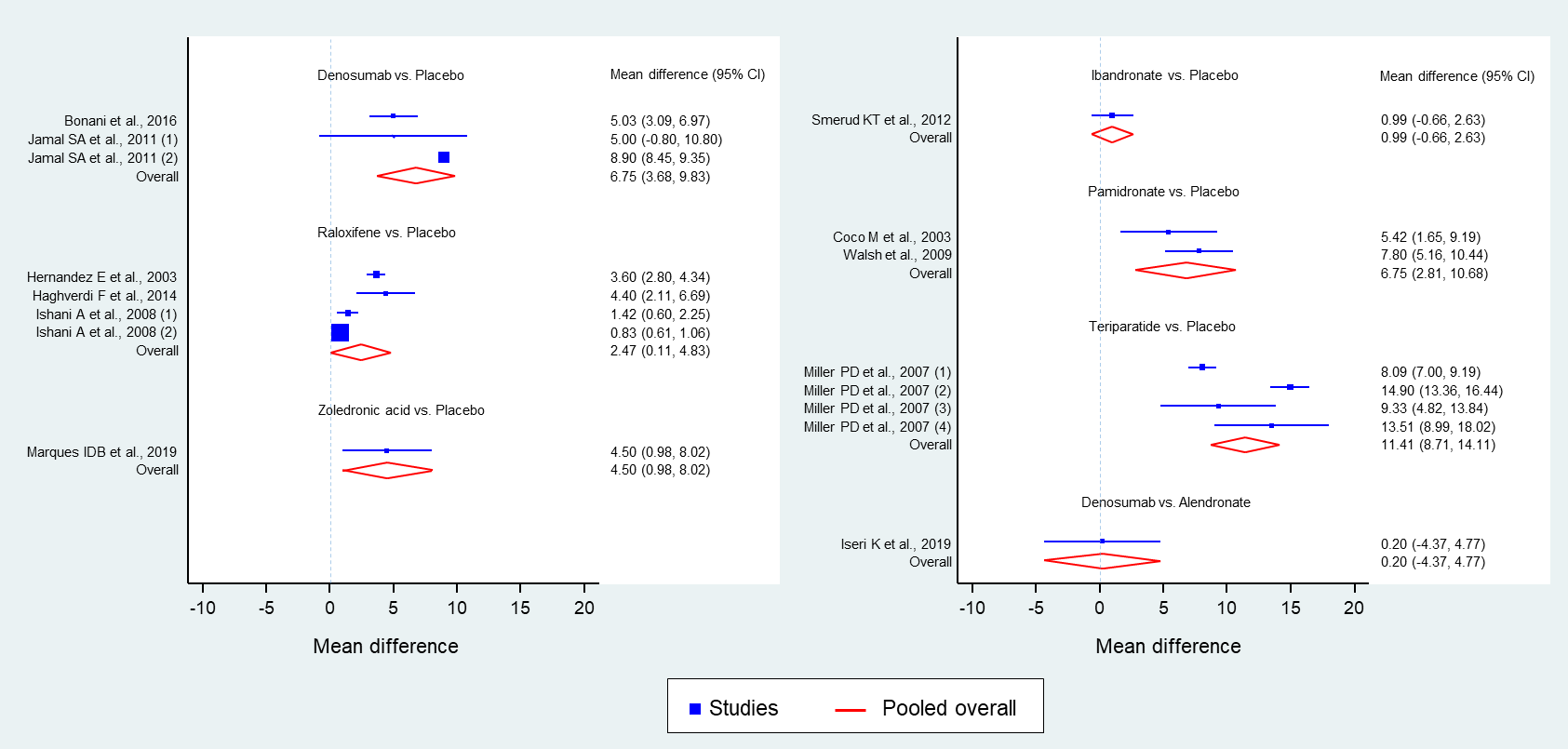
**

**Supplementary Figure 3.** Summary of the head-to-head comparisons of mean difference percentage change of vertebral BMD for the treatment of osteoporosis in patients with CKD or underwent kidney transplantation.

**
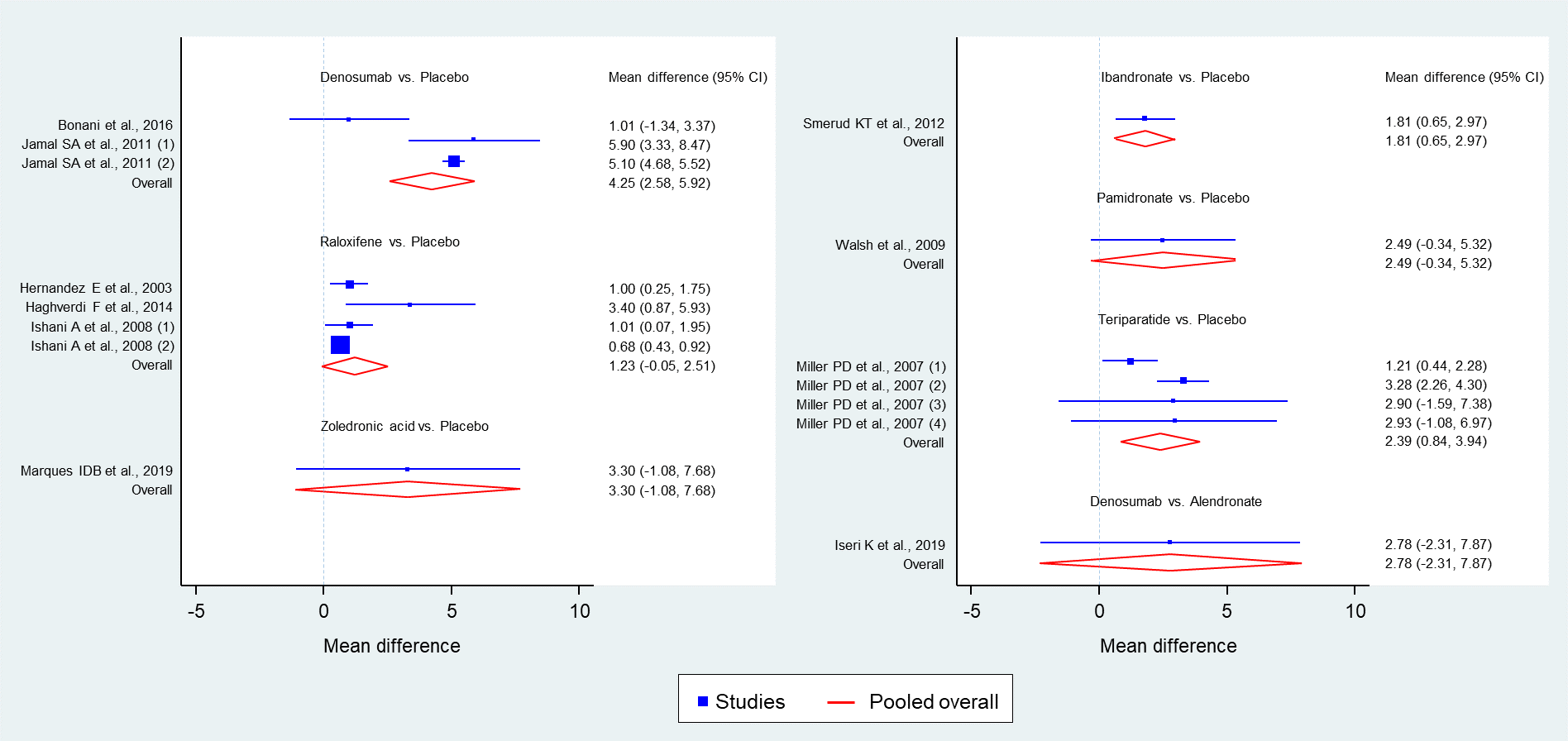
**

**Supplementary Figure 4.** Summary of the head-to-head comparisons of mean difference percentage change of femoral neck BMD for the treatment of osteoporosis in patients with CKD or underwent kidney transplantation.

| Supplementary Tables **Supplementary Table 1.** Summary of the Number of Adverse Events in Selected Randomized Controlled Trials | | | | | |
| --- | --- | --- | --- | --- | --- |
| **Study, Year** | **Death** | **CVD** | **Infections** | **Renal adverse events** | **Musculoskeletal pain** |
| Coco M et al., 2003 | Not reported | Not reported | Not reported | Not reported | Not reported |
| Hernandez E et al., 2003 | Not reported | Not found | Not reported | Not reported | Not reported |
| Jamal SA et al., 2007 | The incidence of death was 1.6% in patients with eGFR<45mL/minute compared with 1.9% in patients with eGFR ≧45mL/minute. | The incidence of CV events was 2.6% in patients with eGFR < 45mL/minute compared with 3.2% in patients with eGFR ≧45mL/minute. | Not reported | The increase in serum creatinine was the same compared with placebo. | Not reported |
| Miller PD et al., 2007 | Not reported | Not reported | Not reported | No significant difference of teriparatide 20mcg or 40mcg daily compared with placebo among patients with GFR 30-80 ml/minute in the incidence of renal-related adverse events. | No significant differences of teriparatide 20mcg or 40mcg daily compared with placebo among patients with GFR 30-80 ml/minute in the incidence of arthralgia. |
| Ishani A et al., 2008 | 11.05% patients with eGFR <60 ml/minute discontinued treatment because of an adverse event or death. | Not reported | Not reported | Not reported | Not reported |
| Walsh et al.,2009 | Not reported | Not reported | Not reported | The acute rejection episodes, non-rejection acute deterioration in kidney function, and serum creatinine level did not differ between the two groups. | Not reported |
| Toussaint ND et al., 2010 | Each group had 1 death episode | Not reported | Not reported | No significant difference compared with placebo in the decline of renal function. | Not reported |
| Torregrosa JV et al., 2010 | One death episode in control group | Not reported | Not reported | Not found | Not reported |
| Jamal SA et al., 2011 | Not found | No significant difference compared with placebo (6.36% vs. 6.37%) | No significant difference compared with placebo (4.43% vs. 3.50%) | There were no significant differences compared with placebo in the change in serum creatinine by stage 3 and 4 of CKD from baseline to year 1 and year 2. From baseline to year 3, a small but significant difference in the change of serum creatinine (<2 mmol/L) by stage 3 of CKD was found compared with placebo. | Not reported |
| Smerud KT et al., 2012 | Three death episodes in placebo group | Not found | *IV Ibandronate vs. placebo*  CMV infection, n (%)  14 (21.2%) vs. 21 (33.3%)  UTI, n (%)  13 (19.7%) vs. 12 (19.0%)  Pneumonia, n (%)  2 (3.0%) vs. 3 (4.8%) | *IV Ibandronate vs. placebo*  Biopsy verified transplant rejections, n (%) 18 (27.3%) vs. 22 (34.9%) Elevated serum creatinine, n (%) 5 (7.6%) vs. 10 (15.9%) Mean change in serum creatinine, mM -2.1±27.7 vs. 4.2±21.6 | Not reported |
| Haghverdi F et al., 2014 | No treatment-related death occurred in both groups. | No deep and superficial  Thrombophlebitis, arteriovenous fistula, graft, dialysis catheter thrombosis, and vasomotor symptoms were reported in both groups. | Not reported | Not reported | Not reported |
| Sánchez-Escuredo et al., 2015 | Not reported | Not reported | Not reported | No significant increasing in serum creatinine was found over the study period for Ibandronate or risedronate groups. | Not reported |
| Bonani et al., 2016 | Not found | Not found | There was a higher number of infection in the denosumab group compared with control group (146 vs 99, p<0.05). | No significant differences compared with control group in the rate of acute rejection (10.9% vs.6.8%), loss of graft function (2.2% vs. 0%) and the change in eGFR. | Leg pain occurred in 9 patients (19.6%) with denosumab group versus 9 patients (20.5%) with control group (p>0.05). |
| Shigematsu et al., 2017 | Not reported | Not reported | Not reported | The incidence of urinary- and kidney function-related adverse events with CKD stage G3 patients was 3.1%. | Not reported |
| Iseri K et al., 2019 | No significant difference in the incidence between denosumab (n=2) and alendronate (n=1) groups. | No significant difference in the incidence of stroke, heart failure and Idiopathic intracranial hypertension between denosumab (n=2) and alendronate (n=3) groups. | No significant difference in the incidence of pneumonia and arteriovenous graft infection between denosumab (n=2) and alendronate (n=1) groups. | Not reported | No significant difference in the incidence between denosumab (n=2) and alendronate (n=2) groups. |
| Marques IDB et al., 2019 | Not reported | Not reported | Not reported | No significant difference in eGFR was found between zoledronate-treated and control groups | Not reported |
| Sugimoto T et al., 2019 | Not reported | Not reported | Not reported | Two persons (4.9%) in risedronate group with CKD stage G3 developed renal and urinary disorders. | Not reported |

| **Supplementary Table 1.** Summary of the Number of Adverse Events in Selected Randomized Controlled Trials (cont.) | | | | | | | |
| --- | --- | --- | --- | --- | --- | --- | --- |
| **Study, Year** | **Gastrointestinal adverse events** | **Hypercalcemia** | **Hypocalcemia** | **Hyperphosphatemia** | **Hypophosphatemia** | **Hypersensitivity reactions** | **Osteonecrosis of the jaw** |
| Coco M et al., 2003 | Not reported | No significant difference between the two groups | No significant difference between the two groups | No significant difference between the two groups | No significant difference between the two groups | Not reported | Not reported |
| Hernandez E et al., 2003 | Not reported | Not reported | Not reported | Not reported | Not reported | Not reported | Not reported |
| Jamal SA et al., 2007 | The incidence of GI events was 4.5% in patients with eGFR < 45mL/minute compared with 5.2% in patients with eGFR ≧45mL/minute. | Not reported | Not reported | Not reported | Not reported | Not reported | Not reported |
| Miller PD et al., 2007 | Not reported | There was significant difference by the incidence of hypercalcemia of teriparatide 20mcg/day or 40mcg/day among patients with GFR 30-80 ml/minute compared with placebo. | Not reported | Not reported | Not reported | Not reported | Not reported |
| Ishani A et al., 2008 | Not reported | Not reported | Not reported | Not reported | Not reported | Not reported | Not reported |
| Walsh et al., 2009 | Not reported | Not reported | Five patients (8.6%) experienced transient hypocalcemia in pamidronate group. | Not reported | Not reported | Not reported | Not reported |
| Toussaint ND et al., 2010 | One patient in alendronate group developed GI adverse events. | No significant difference between the two groups | No significant difference between the two groups. | Not reported | No significant difference between the two groups | Not reported | Not reported |
| Torregrosa JV et al., 2010 | Dyspepsia occurred in one patient of risedronate group. | Hypercalcemia occurred in one patient of risedronate group. | Not reported | Not reported | Not reported | Not reported | Not reported |
| Jamal SA et al., 2011 | Not reported | Not reported | Not reported | Not reported | Not reported | Not reported | Not reported |
| Smerud KT et al., 2012 | Gastroenteritis occurred in 1 patient (1.5%) of ibandronate group versus 6 patients (9.5%) of placebo group (p>0.05). | Hypercalcemia occurred in 11 patients (16.7%) of ibandronate group versus 13 patients (20.6%) of placebo group (p>0.05). | Not reported | Not reported | Not reported | Not reported | Not reported |
| Haghverdi F et al., 2014 | No dyspepsia reported in both groups. | Not found. | Not found. | Not found. | Not found. | Not reported | Not reported |
| Sánchez-Escuredo et al., 2015 | Five patients in risedronate group developed GI intolerance. | No significant difference between the two groups | No significant difference between the two groups | Not reported | Not reported | Not reported | Not reported |
| Bonani et al.,  2016 | More diarrhea events occurred in denosumab group (50.0%) comparing control group (29.5%) with significant difference (p < 0.05). | Less frequent episodes of hypercalcemia (>2.6 mmol/L) in the denosumab group (37 vs. 55). | More frequent episodes of asymptomatic and transient hypocalcemia (<1.9 mmol/L) in the denosumab group (12 vs. 1). | Not reported | Not reported | Not reported | Not reported |
| Shigematsu et al., 2017 | The incidence of GI symptoms–relate adverse events with CKD stage G3 patients was 38.6%. | Not found. | Not found. | Not found. | Not found. | Not reported | Not found. |
| Iseri K et al., 2019 | Two episodes of ileus and cholecystitis happened in alendronate group. | Higher incidence of hypercalcemia in alendronate group (37.5%) during the first 2 weeks compared with denosumab group (9.1%) with significant difference (p<0.05). | Higher incidence of hypocalcemia in denosumab group (27.3%) during the first 2 weeks compared with alendronate group (4.2%) with significant difference (p<0.05). | No significant difference between the two groups | No significant difference between the two groups | Not reported | Not reported |
| Marques IDB et al., 2019 | Not reported | Not found. | Not found. | Not found. | Not found. | Not reported | Not reported |
| Sugimoto T et al., 2019 | The incidence of GI disorders was range from 2.4 to 41.5%. | Not found. | Not found. | Not found. | Not found. | Not reported | Not found. |
